# On the Employment of Tepid Baths in Diseases of the Chest, Especially in Pulmonary Phthisis

**Published:** 1875-03

**Authors:** 


### ON THE EMPLOYMENT OF TEPID BATHS IN DISEASES OF THE CHEST, ESPECIALLY IN PULMONARY PHTHISIS.

It is an opinion generally held by the public, and even by physicians, that baths are injurious to patients suffering from any pulmonary complaint, and it is considered heroic treatment in hospital, and, above all, in private practice, to presricbe a bath to a patient who coughs, even when its administration is indicated for the relief of other complaints as, for instance, urethral stricture, etc. Tepid and hot baths are recommended in a large number of diseases; and all authors, who have observed their action on man in health, agree in extolling their excellent effects. Regarding the judiciousness of their employment in diseases of the chest, there is also no question, though hitherto but few scientific observations have been made which would favor this view of their efficacy. Dr. Sonplet, at La Pitie, in the service of Prof. Lasèque, has instituted a series of experiments, the results of which permit him to conclude that, by discarding the employment of tepid baths in thoraric maladies, we deprive ourselves of an important and incontestable aid in the treatment. The author bases his conclusions on two hundred observations, most of the baths having been administered to consumptives. In administering a bath, its temperature is the most important point, the occasional addition of a small quantity of sulphuret of potassium, alkaline carbonates, etc., being all secondary; thermometric indications do not always correspond to the sensations of heat and cold, and the physiological and pathological condition of the subjects must be taken into account, and also their individual impressibility. A *tepid* bath is one the temperature of which is about three degrees below that of the body; the temperature determines that of the bath; from time to time more water is added, to allow of no variation. The duration of a bath varies from twenty to forty-five minutes; the patient is allowed to remain in it until he feels fatigued, or chilled even in warm water. There is no indication to discontinue the bath provided the patient feels comfortable in it; but the desire to remain is rarely prolonged beyond forty-five minutes, even in summer. On account of ths increased appetite which is felt after a bath, the hour which precedes the morning or evening repast is the one which agrees best with patients. Usually, one bath is given every two days. A powerful influence is immediately exerted on the sweats, of which the patients complain so much; the baths are, however, increased to one bath every day when the effect is not sufficiently notable from the very beginning of the treatment; rarely do the sweats resist the third or fourth bath. Afterward, two or three baths a week suffice to check them, and to bring about a marked amelioration, especially in cases of less advanced and less extensive tuberculous disease. This treatment is only accessory, the general treatment being continued, though no anti-sudorifics have been given in the above cases. During the first two or three baths, the patients generally feel a slight oppression acceleration of the respiratory movements, lasting from two to five minutes, which disappears in the third or fourth bath. In patients who have continuous cough this disappears, and the expectoration becomes more copious; the pulse diminishes its frequency, and the temperature falls. On leaving the bath, the patient feels comfortable, respires more easily, the appetite is increased, the skin has more tone, the pulse is diminished in frequency; the author has seen it reduced twelve, twenty and twenty-eight pulsations per minute; the temperature is sometimes reduced two degrees. In cases of acute phthisis this amelioration does not last more than three or four hours; but during that time the patients are materially alleviated. In consumptives the skin is in an habitual state of moisture, which softens it, and makes it pale and inelastic; in a word, it loses its vitality and a portion of its respiratory faculties. The bath tonifies it, restores its capillary circulation, cleanses it, and removes everything which embarrasses it in the performance of its functions. Pulmonary respiration being very incomplete, the complementary respiration by the skin plays an important *rolê* in hematosis, in facilitating the exchange of gases. In many cases associated with diarrhœa this has been amended by the use of baths; the presence of this complication is, therefore, no contraindication to their employment. Contrary to the opinion of Fröhlich, that the temperature of the bath should be in inverse ratio to that of the subject, the author believes with Ziemssen that a temperate bath continued thirty or forty minutes, produces a more prolonged abatement of febrile heat than a cold bath of ten minutes.—*Arch. Gén. de Méd. Rev. Therapeut.*

